# The contribution of seed dispersers to tree species diversity in tropical rainforests

**DOI:** 10.1098/rsos.150330

**Published:** 2015-10-14

**Authors:** Satoshi Kakishima, Satoru Morita, Katsuhiko Yoshida, Atsushi Ishida, Saki Hayashi, Takahiro Asami, Hiromu Ito, Donald G. Miller, Takashi Uehara, Shigeta Mori, Eisuke Hasegawa, Kenji Matsuura, Eiiti Kasuya, Jin Yoshimura

**Affiliations:** 1 Graduate School of Science and Technology, Shizuoka University, Hamamatsu, Shizuoka 432-8561, Japan; 2 Department of Mathematical and Systems Engineering, Shizuoka University, Hamamatsu, Shizuoka 432-8561, Japan; 3 Biodiversity Conservation Planning Section, Center for Environmental Biology and Ecosystem Studies, National Institute for Environmental Studies, Tsukuba, Ibaraki 305-8506, Japan; 4 Center for Ecological Research, Kyoto University, Otsu, Shiga 520-2113, Japan; 5 Department of Biology, Shinshu University, Matsumoto, Nagano 390-8621, Japan; 6 Department of Biological Sciences, California State University, Chico, CA 95929, USA; 7 Department of Food, Life, and Environmental Science, Faculty of Agriculture, Yamagata University, Tsuruoka, Yamagata 997-8555, Japan; 8 Department of Ecology and Systematics, Graduate School of Agriculture, Hokkaido University, Sapporo, Hokkaido 060-8589, Japan; 9 Laboratory of Insect Ecology, Graduate School of Agriculture, Kyoto University, Kyoto 606-8502, Japan; 10 Department of Biology, Faculty of Science, Kyushu University, Fukuoka 812-8581, Japan; 11 Marine Biosystems Research Center, Chiba University, Kamogawa, Chiba 299-5502, Japan; 12 Department of Environmental and Forest Biology, State University of New York College of Environmental Science and Forestry, Syracuse, NY 13210, USA

**Keywords:** animal seed dispersers, coexistence, glacial periods, multi-layer lattice model, tropical rainforests

## Abstract

Tropical rainforests are known for their extreme biodiversity, posing a challenging problem in tropical ecology. Many hypotheses have been proposed to explain the diversity of tree species, yet our understanding of this phenomenon remains incomplete. Here, we consider the contribution of animal seed dispersers to the species diversity of trees. We built a multi-layer lattice model of trees whose animal seed dispersers are allowed to move only in restricted areas to disperse the tree seeds. We incorporated the effects of seed dispersers in the traditional theory of allopatric speciation on a geological time scale. We modified the lattice model to explicitly examine the coexistence of new tree species and the resulting high biodiversity. The results indicate that both the coexistence and diversified evolution of tree species can be explained by the introduction of animal seed dispersers.

## 1. Introduction

Tropical rainforests are known for their extreme angiosperm diversity [1,2]: their biological richness exceeds that of any other plant community. Beginning in the 1970s, many hypotheses have been advanced to explain this high diversity [3–6]. Some of the hypotheses are based on ecological factors, such as seed predators, pathogens, seed banks, pollinators, light conditions, ant–plant interactions and seedling survival [7–23]. Among them, the negative density dependence of seedlings demonstrates the importance of seed predation, pathogens and seedling survival [19–24]. Other hypotheses reflect the statistical properties of migration and extinction [6,25,26]. Although there are many plausible causes of the extreme tree diversity of tropical rainforests, we still have no conclusive explanation of the mechanisms for the coexistence of so many tree species in tropical rainforests [1–3,6,26–28].

Among the ecological factors affecting tree species diversity, the importance of biotic interactions has been noted, especially mutualistic interactions [17,29–32]. Mutualism is a characteristic feature of tropical rainforests. Most tree species in tropical rainforests are angiosperms known to produce nectar and pollen in flowers attractive to insect and vertebrate pollinators and to bear fruit eaten by seed-dispersing animals [2]. Many intriguing cases of tight species-to-species mutualism are known in insects [8–10,29,30–34], e.g. a fig tree and its fig wasp, and orchids and their pollinators; the effects of pollinators and ant–tree interactions have been considered theoretically [18,33]. Angiosperm trees have been found to have relatively close relationships with specific seed dispersers, notably birds, bats and some other mammals [35–37], in mutualisms characterized as dispersal syndromes [38]. For example, some tree species yield a hard seed that can be broken and germinated only by elephants [39]. The importance of seed dispersers for the coexistence of tree species is well established [35–37,40,41]. Thus, the diversity of angiosperm tree species may originate from and is maintained by the mutualistic relationships of tree species and their pollinators and seed dispersers. Recent models explore the contribution of seed dispersers to tree diversity [42–46]. Here, we incorporate animal movements (paths) into a lattice space model.

We propose that the present extremely high diversity of tree species in tropical rainforests originates from tight mutualisms among angiosperm trees and their specific animal seed dispersers. In our model, we introduce species-specific animal movements for seed dispersal. These movements reduce competition among tree species. Because each animal species has a unique movement pattern [47–49], the dimensions of its habitat become uniquely species-specific [50,51]. Therefore, we may find an animal in one location, but never in another, even if the required habitat components are the same [52].

Geologically, tropical rainforests have experienced repeated fragmentations due to climatic oscillations [2,53–57]. Milankovich cycles and changes in the composition of the atmosphere have resulted in alternating glacial and interglacial periods, causing sea-level oscillations over approximately 120 m during the Cenozoic Era [58]. As a result, since the beginning of the Cenozoic Era, land areas (in particular, those supporting tropical rainforests) have been repeatedly fragmented by increasing sea levels during warm interglacial periods, then reunited by decreasing sea levels during cool glacial periods. Even on large continents such as those on which the Amazon and the African tropics occur, savannah communities predominated during glacial periods because of a decrease in precipitation [2,29,56]. During the glacial periods, rainforests were evidently separated and confined to small areas of each continent. These processes promoted speciation in many angiosperm tree lineages as well as in birds and insects. High species diversity in a community implies highly frequent speciation events and less frequent extinctions [57,59,60]. In this study, our models consider frequent allopatric speciation events due to repeated separations and reunifications of habitats caused by intermittent ice ages [61]. Allopatric speciation is assumed to have occurred in every mutualistic pair of a tree species and its partner. For example, if a single habitat were separated into three isolated areas twice, the total number of species would be 3^2^=9 tree species [57]. If a habitat were separated four times, then the total is 3^4^=81 tree species. Hence frequent geographical fragmentation is suggested as a core mechanism producing the high species diversity in many rainforest taxa [53,61].

Here, we build a lattice simulation model to incorporate the role of animal seed dispersers in the coexistence of tree species. We investigate whether the movements of animal seed dispersers affect tree species diversity and what characteristics of the movement of animals are important for the coexistence of tree species. In our basic model, we evaluate the effects of seed dispersers on the coexistence of tree species. In the extended evolutionary model, we incorporate the traditional theory of allopatric speciation by repeated fragmentation on a geological time scale to examine the promotion of species diversity.

## 2. Material and methods

### 2.1 Lattice model framework

**Figure 1.**
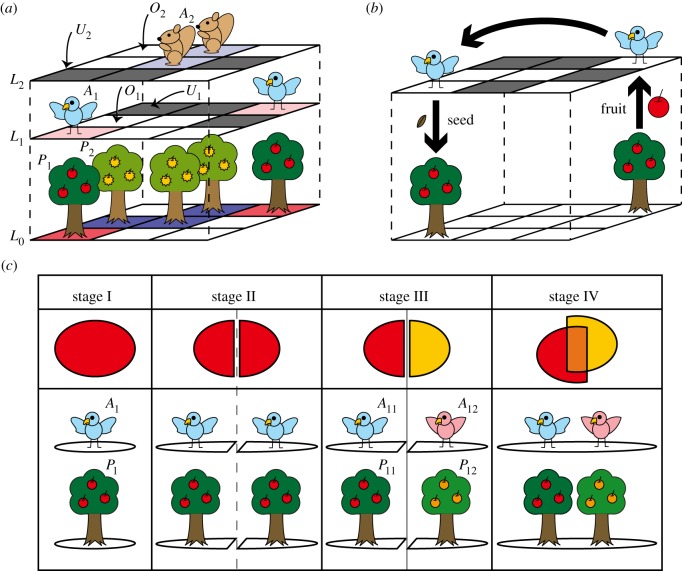
Schematic diagram of the population and evolution models. (*a*) Schematic diagram with two tree species (*P*_1_ and *P*_2_ on plant layer *L*_0_) and the corresponding seed dispersers (birds *A*_1_ on layer *L*_1_ and squirrels *A*_2_ on *L*_2_). In each animal layer, the grey cells (*U*_1_ and *U*_2_) indicate unvisited sites (where animals never visit). (*b*) An animal (bird) eats a fruit (apple) at the tree (*P*_1_) and drops a seed at an unoccupied site (*O*_1_) that results in a new apple tree. (*c*) The fragmentation process repeats at every 6000 MCS, such that (stage I: a single habitat) →3000 MCS→ (stage II: habitat separation and stage III: species differentiation) →3000 MCS→ (stage IV: habitat reunification and resulting species coexistence=stage I).

Focusing on the mutualism between seed plants and their seed dispersers, we built a multi-layer lattice model of tree species with animal seed dispersers (figure 1*a*,*b*). All tree species grow in the plant layer of the lattice, while each animal has its own species-specific habitat and disperses the seeds of its partner within its habitat range in its own lattice layer. We assume species-specific patterns of seed dispersal by animal partners reduce interspecific competition among tree species, promoting their coexistence.

To verify the coexistence and persistence of tree species, we first built a (*n*+1) multi-layer lattice ecosystem model with *n* tree species (*P*_*i*_ for *i*=1, 2,…,*n*) and their corresponding seed dispersers (*A*_*i*_ for *i*=1,2,…,*n*; figure 1*a*,*b*). The lattice size is 200×200 cells. The first layer is a plant layer (*L*_0_), in which all tree species grow, and each animal species (*A*_*i*_) moves within its own layer (*L*_*i*_ for *i*=1, 2,…,*n*) to disperse seeds (figure 1*a*). Each cell in the plant layer (*L*_0_) is either occupied by the *i*th tree species (*P*_*i*_) or unoccupied (vacant: *O*_0_). The seeds of a plant (*P*_*i*_) are carried only by its animal partner (*A*_*i*_) (figure 1*b*). The cells in an animal layer of the lattice are classified into two types: (i) cells where animals of species *i* can visit (either occupied or vacant: *A*_*i*_ or *O*_*i*_, respectively) and (ii) cells where animals of species *i* cannot visit (unvisited site: *U*_*i*_).

We evaluated the overall effects of the unvisited cells by varying their proportion in the animal lattice layers. As a control, we also built a single-layer lattice model without animals, where seeds were dispersed globally.

### 2.2 Specifications for the population model

To explore specifically the effects of animal–plant interactions on tree diversity, we introduced the following simplifications: animals are immortal with no reproduction, flowers are automatically pollinated to produce seeds and, after a seed is carried to a vacant cell, it will automatically grow to an adult tree, unless it dies (with a constant risk of mortality).

The process of seed dispersal and reproduction is as follows. In an animal layer (*L*_*i*_), we pick a cell randomly. If the cell is occupied by an animal (*A*_*i*_), we randomly choose one vacant cell (*O*_*i*_) and move the animal to that cell, i.e. *A*_*i*_+*O*_*i*_→*O*_*i*_+*A*_*i*_ (figure 1*b*). When the animal *A*_*i*_ moves, it carries and disperses the *P*_*i*_ seed if the animal was located above the cell where the *P*_*i*_ plant grows. If the seed is carried into a vacant cell, it automatically grows to an adult tree, i.e. *P*_*i*_+*O*_*i*_→*O*_*i*_+*P*_*i*_ (figure 1*b*). Definitions of our symbols are given in table 1.

**Table 1.** Glossary of symbols.

symbol	description
*P*_*i*_	plant growth sites (cells). The subscript denotes species
*A*_*i*_	sites visited by animals (cells). The subscript denotes species
*L*_*i*_	layers. *L*_0_: a plant layer; *L*_1_–*L*_*n*_: animal layers for *A*_1_–*A*_*n*_
*U*_*i*_	unvisited sites (cells). The subscript denotes species
*O*_*i*_	unoccupied sites (cells). The subscripts denote either a layer, animal species, or both
*u*_*i*,_	unvisited rates. The subscript denotes species
*m*_*i*_	mortality rates. The subscript denotes species
*v*	niche-shift probability

We varied the unvisited rates *u* of a plant species as follows. In the initial simulations, we set the unvisited rate *u* to be the same for all species. We varied the constant unvisited rate between 0 and 1 to examine its effects on the number of coexisting species. We also varied the unvisited rates of species to examine the superiority of species among species, such that *u*_*i*_=1.0−0.1*i*. We also considered the case when seed-dispersal mutualisms are not perfectly species-specific. When animal *A*_*i*_ moves to the cell where the *P*_*j*_ ( *j*≠*i*) plant grows, it carries and disperses the *P*_*j*_ seed with a decreased probability (20% or 50%).

A randomly chosen plant also dies at a constant mortality rate *m*_*i*_, i.e. *P*_*i*_→*O*_*i*_. Here, we make the species-specific mortality rates differ among species such that *m*_*i*_=0.11−0.01*i* for *i*=1,…,8. The reason for this difference among species is to expedite simulation results. If we set the same mortality rate among all species, the simulation generates random walks in which the convergence time becomes excessively long. This species difference also makes the coexistence of species much less likely in the context of competitive interactions. We also examined the effects of mortality on the number of coexisting species and their density. Here, we keep the mortality of all species identical (the basic mortality *M*=*m*_*i*_ for *i*=1,…,8), and the unvisited rates are varied among species such that *u*_*i*_=1.0−0.1*i* for *i*=1,…,8. We performed this procedure for a given number of (Monte Carlo) steps. We set each Monte Carlo step (MCS) as 200×200 times. The initial densities of *A*_*i*_ and *P*_*i*_ are 0.1 for all population simulations. The template of a simulation program for the population model is provided in the electronic supplementary material, text S1.

### 2.3 Specifications for the evolutionary model

To simulate the evolutionary diversification of tree species, we applied repeated separations and unifications of habitats in the lattice model, where the separated trees and animal partners were assumed to evolve a new mutualistic relationship. The diversification of tree species was evaluated by the same lattice model by introducing repeated geographical isolations (figure 1*c*). The procedure consists of four stages: (I) the initial single-lattice habitat; (II) two isolated habitats; (III) the species differentiation stage, where each tree–animal system has diverged into two independent systems; and (IV) the habitat unification stage, where the isolated habitats are reunited into a single large habitat (=stage 1). This process approximates the evolutionary diversification of trees and their associated seed dispersers during repeated fragmentation by glacial periods on a geological time scale. A single large habitat before glaciation episodes (I) becomes isolated into two small isolated habitats when glaciation begins (II). During the glacial period (III), trees and their associated seed dispersers diverge into two distinct species. After the glacial period (IV), the two isolated habitats are united, owing to global warming during the following interglacial period. Geologic evidence indicates this process was repeated over many glacial periods during the Cenozoic Era [62], yielding the diversification of many animal and plant taxa [53,63,64]. We then evaluated the number of surviving species after the repeated fragmentations and unifications of the lattice space.

All the combinations of trees and animal dispersers are doubled during stage III when the habitat is separated. At stage II, the entire lattice is divided in half (200×100 cells). At stage III, we introduce speciation in all tree–animal interactions, i.e. a partnership (*P*_*i*_−*A*_*i*_ for *i*=1,2,…,*n*)→two independent partnerships (*P*_*ij*_−*A*_*ij*_ and *P*_*ij*_−*A*_*ij*_ for *i*=1,2,…,*n* and *j*=1,2, where *j* denotes an isolated habitat). Stage IV becomes Stage I of the next speciation cycle. After speciation begins, habitat modification occurs such that when the lattice space is divided in half, the distribution of the unvisited sites *U*_*i*_ is modified into two different distributions (*U*_*i*1_ and *U*_*i*2_) in the following manner. The animals *A*_*ij*_ are resident in each half lattice (*j*=1,2), the unvisited cells *U*_*ij*_ in the *j*-lattice remain unmodified, while the unvisited cells *U*_*ik*_ (*k*≠*j*) in the *k*-lattice are moved to its neighbouring cells with a niche-shift probability (*v*: described below), such that *U*_*ij*_+*O*_*ij*_→*O*_*ij*_+*U*_*ij*_. Here, we modify the neighbouring cells in particular, because the niche shift of new species differs only slightly from habitat preferences compared with parental species (phylogenetic niche conservatism) [65,66]. If the selected cell is either an already unvisited cell or outside the boundary, movement to the cell is negated. If the niche-shift probability *v*=0, then no speciation takes place, while a non-zero *v* means speciation with niche shift from the original habitat (visited cells). We set *v* as the same value among all species, assuming speciation is synchronized between trees and seed dispersers. Biotic interactions, such as host–mutualist and host–parasite interactions, are known to yield synchronized divergence patterns [34,67,68]. To explore temporal dynamics, we set *v*=10^−2^ for the initial simulation. To evaluate the effects of *v* and *u*, we set *v* between 10^−2^ and 10^−5^ for the current simulations. We varied the unvisited rates *u* of a species, as follows. In the initial evolutionary simulations, we set the unvisited rate *u*=0.8 for all species. We also varied the constant unvisited rate between 0 and 1 to examine its effects on the number of coexisting species. When we evaluated the effects of niche-shift probability *v*, we set *u*=0.8, 0.6 and 0.4.

We start with three species and this fragmentation process is repeated three times: three plant species (*P*_*i*_ for *i*=1,2,…, *n*, where *n*=3). Therefore, the total number of coexisting species if no extinction takes place becomes 3×2=6 species (*P*_*ij*_ for *j*=1, 2) in a single iteration, 6×2=12 species (*P*_*ijk*_ for *j*, *k*=1,2) for two iterations, and 12×2=24 (*P*_*ijkl*_ for *j*,*k*,*l*=1,2) for three iterations. Initially, we set the species-specific mortality rates of three species, such that *m*_*i*_=0.032−0.02*i* for *i*=1, 2, 3 and all the derived species are assigned the same mortality rate as the parental species. The initial densities of *A*_*i*_ and *P*_*i*_ were 0.2 for all evolutionary simulations. The template of a simulation program for the evolutionary model is provided in the electronic supplementary material, text S2.

## 3. Results

**Figure 2.**
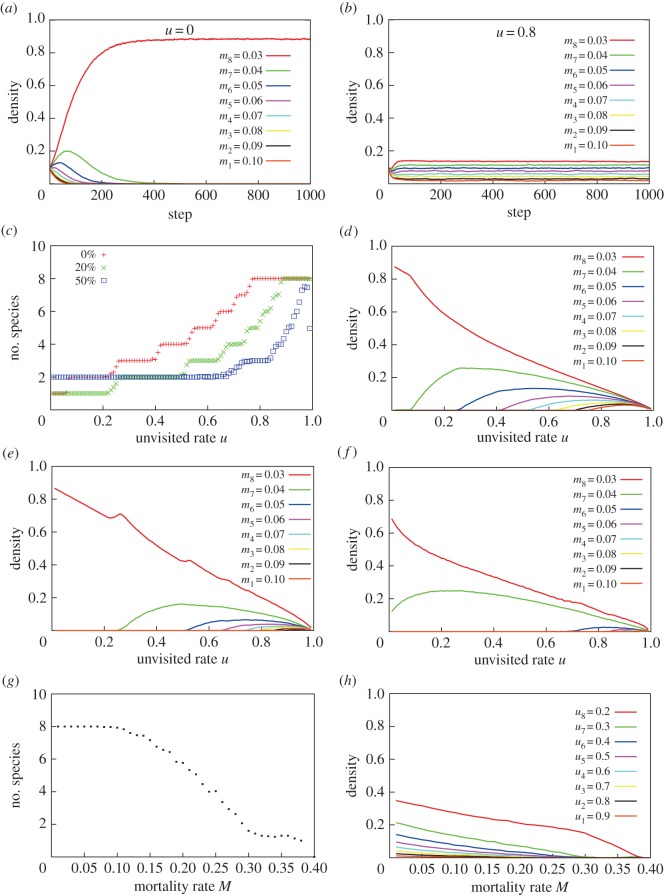
Persistence of eight tree species in the multi-layer lattice model of tropical rainforests. (*a*) Dynamics with no unvisited sites (unvisited rate: *u*=0). (*b*) Dynamics with a high unvisited rate (*u*=0.8). (*c*) The number of surviving species at steady state against *u*. (*d*–*f*) Steady-state densities against *u*, when the animal *A*_*i*_ carries and disperses the *P*_*j*_ ( *j*≠*i*) seed with (*d*) 0%, (*e*) 20% or (*f*) 50% probability. (*g*) The number of surviving species as a function of mortality rate *m*_*i*_=*M*. (*h*) Steady-state densities against *M*. (*a*–*f*) Mortality rates of individual species are *m*_*i*_=0.11−0.01*i*. (*c*–*h*) Mean values of 30 trials. Steady-state density is estimated at 3000 MCS, because all simulation runs reach a steady state by 1000 MCS. (*g*,*h*) Unvisited rates of individual species are *u*_*i*_=1.0−0.1*i*. (*a*–*h*) The initial densities of *A*_*i*_ and *P*_*i*_=0.1. The drawing was created by S.H. and J.Y.

The coexistence and persistence of species diversity was verified using a (8+1) multi-layer lattice ecosystem model with eight tree species and their corresponding eight animal species for seed dispersal (figure 2). When there were no unvisited sites (*u*=0), the tree species with the lowest mortality (*m*_8_=0.03) excluded all other species immediately, occupying 80% of the lattice space (figure 2*a*). By contrast, when 80% of the lattice space consisted of unvisited sites (*u*=0.8), all the tree species coexisted with less than a 0.2 steady-state density (figure 2*b*). In this case, the steady-state density of species depended on the mortality rate, such that superior species (species with a lower mortality rate) occurred at high densities. Thus, coexistence of two or more tree species was nearly impossible when tree seeds were randomly dispersed (*u*=0), while coexistence was guaranteed when the dispersal of tree seeds were highly restricted due to limited animal movements (*u*=0.8). When seeds were dispersed globally without animals, the tree species with the lowest mortality rate (*m*_8_=0.03) excluded all other species, which also occurred with animal dispersal with no unvisited sites (*u*=0) (electronic supplementary material, figure S1).

We plotted the number and densities of tree species against the rates at which they were not visited (‘unvisited’) (figure 2*c*,*d*). When the unvisited rate increased, the number of coexisting species increased as a stepwise function, totalling eight species at about *u*=0.8 (figure 2*c*). For the density of species, when the unvisited rate increased, the density of already existing species decreased, while newly coexisting species increased in density (figure 2*d*). When *u*=0.75−0.99, all eight species coexist with very low densities.

When the species specificity of seed dispersal is lower, the number of coexisting species decreases compared with perfect species-specificity (figure 2*c*). However, when the unvisited rate is high, the results are the same if animals can disperse the seeds of other plant species with a 20% probability. Even if this probability is increased to 50%, the number of coexisting species converges when the unvisited rate approaches unity. Thus, this mechanism is highly effective even if animal dispersers exhibit diminished species-specificity.

We plotted the number and densities of tree species against the basic mortality rates *M* (identical for all eight species), while the unvisited rates varied among species (*u*_*i*_=1.0−0.1*i* for *i*=1,…,8). When the basic mortality rate was low (*M*<0.1), all eight species coexisted (figure 2*e*). When mortality rates were further increased, the number of surviving species decreased rapidly, reaching only one species at approximately *M*=0.3. As the basic mortality rate *M* increased, the densities of all species decreased (figure 2*f*). Here, a superior species remaining at a higher steady-state density always had a lower unvisited rate. Thus, species with a low unvisited rate always maintained a high density. However, coexistence became impossible when all animals moved freely to disperse seeds, i.e. *u*=0 (figure 2*c*,*d*), or when the mortality rate was too high (figure 2*e*,*f*).

**Figure 3.**
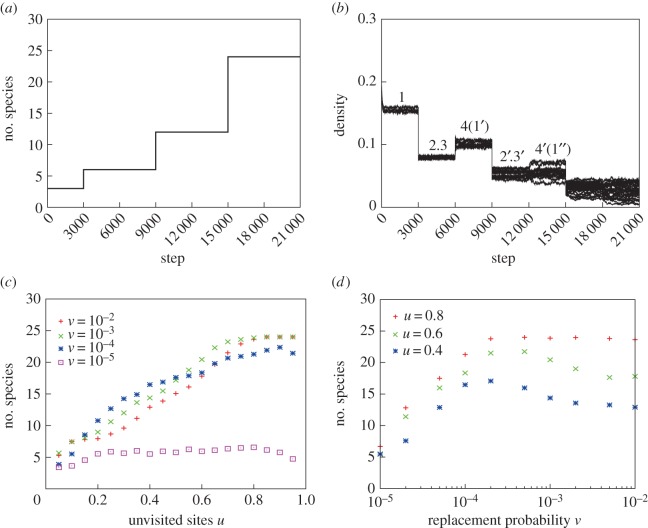
Diversification model via repeated habitat fragmentation in the multi-layer lattice model of tropical rainforests. (*a*) Dynamics of species diversity. (*b*) Density dynamics of all species. (*c*) The final number of species against the unvisited rate *u* for various levels of shift rate *v* in unvisited sites. With probability *v*, an unvisited site is shifted to a neighbouring cell (*v*=10^−2^, 10^−3^, 10^−4^ and 10^−5^). (*d*) The final number of species against the probability *v* for various *u* (=0.4, 0.6 and 0.8). (*c*,*d*) The averages of 30 trials were measured at 21 000 MCS. Parameters are: *m*_1_=0.03, *m*_2_=0.028 and *m*_3_= 0.026 and the initial densities of *A*_*i*_ and *P*_*i*_=0.2. The drawing was created by S.H. and J.Y.

Figure 3 shows the simulation results for the diversified evolutionary model. Figure 3*a* shows the temporal dynamics of the number of coexisting species starting from three species (*m*_*i*_=0.11−0.01*i*, for *i*=1, 2, 3), all with a 0.2 initial density with the unvisited rate *u*=0.8 and the niche-shift probability *v*=10^−2^. With speciation events, the total number of coexisting species reached 24 species with no extinctions (figure 3*a*). The temporal dynamics of densities (figure 3*b*) showed a large reduction in density when the lattice space was fragmented into two halves because the number of species doubled (Stage II and III). Accordingly, when the fragmented lattices were reunited, the density of each species was increased.

We plotted the number of surviving species against the unvisited rate *u* for four different levels of the niche-shift probability *v* (10^−2^, 10^−3^, 10^−4^ and 10^−5^) (figure 3*c*). When the niche-shift probability *v* was relatively large (*v*=10^−2^−10^−3^), no extinction occurred; this resulted in the maximum of 24 species as long as the unvisited ratio was high (*u*>0.8). However, when *v* was smaller (*v*=10^−4^), extinctions occurred, resulting in fewer than 24 species, even if the unvisited ratio was sufficiently high (*u*>0.8). Furthermore, when *v* was too small (*v*=10^−5^), the number of surviving species became close to the initial number of species.

To extend our analysis, we plotted the number of surviving species against the niche-shift probability *v* for three different levels of the unvisited rate (0.4, 0.6 and 0.8) (figure 3*d*). These plots reveal an optimal level of the niche-shift probability (*v*), indicating that the behavioural pattern of animals is critical for the coexistence of tree species; this trend became evident when *u*=0.4 and 0.6.

## 4. Discussion

Our results indicate that the existence of habitat patches not visited by animal dispersers has an important role in the coexistence of species (figure 2). If there are no unvisited patches (*u*=0), animals can move into any sites and deposit the seeds they carry, which is equivalent to random dispersal. In this case, one superior species immediately drives the others to extinction (figure 2*a*). By contrast, if there are many unvisited patches, some sites are likely to be almost exclusively available to one plant species, because of the limited access other species-specific animal dispersers have to these sites. In the extreme case, some sites are available to one animal species only, as the remaining animal species fail to visit these sites. Thus, each tree species occupies specific sites unavailable to the other tree species. Interestingly, the complete exclusion of seed dispersal appears unnecessary to promote coexistence of tree species. It is unknown how many species can coexist in the 40 000 sites (200×200 cells), but at least 24 species are possible in the current simulations (figure 3*a*).

Our model assumes many strict conditions, which may limit its general applicability to tropical rainforests. For example, our model assumes an exclusive one-to-one relationship between a tree species and its seed disperser. In reality, frugivorous animals are often considered generalists [69,70]. Because animals eat many types of fruit, they are likely to carry the seeds of other plants, albeit with lower probabilities. To explore this possibility, we ran the simulation with lowered relationship specificities. The results show that the coexistence of many species is still highly maintained, in spite of lower species specificity in seed dispersal (figure 2*c*). However, there remains a large gap between our assumptions and actual scenarios with frugivores.

Coexistence of multiple species, in our models, is achieved under the following necessary conditions: (i) there are mutualistic seed dispersers with specific movements (resulting in unvisited sites); (ii) the entire ecosystem (here, the whole lattice space) is divided and united repeatedly; (iii) when the ecosystem is divided into two or more patches, speciation occurs in both trees and their associated animal dispersers; and (iv) the changes in behavioural patterns and resulting dispersal sites should be neither too small nor too large. If the niche shift is too small, competitive exclusion takes place involving the parental species. If the niche shift is too large, then the unvisited sites of new animal species are distributed randomly. Thus, slight deviations from niches of the parental species seem important to promote coexistence of closely related species.

There is strong evidence that the fragmentation of distribution ranges results in the loss of diversity [71], probably because the likelihood of extinction increases when the ranges are small. Plant population sizes sufficient to avoid extinction are rather small in our models because the persistence of tree species is guaranteed by seed dispersers (figure 2). In our evolutionary model, fragmentation does not cause the extinction of all species because mutualistic seed dispersers prevent the competitive exclusion of tree species through the animals' unique dispersal behaviour (figure 3*a*,*b*). Moreover, the number of species may be increased exponentially by repeated fragmentation events.

Our hypothesis for extreme diversity in tropical rainforest trees incorporates two critical components: (i) mutualisms between animal seed dispersers and angiosperms and (ii) repeated geographical isolations during the Pleistocene. These elements occur in a unique historical context, where tropical rainforests are restricted to equatorial regions during glacial maxima [54,55]. While few hypotheses for rainforest tree diversity consider the importance of the historical backdrop, our hypothesis rests on assumptions explicitly addressing the biogeographic elements common to tropical rainforests generally [72].

Although our models provide theoretical support for the hypotheses that dispersal syndromes and range contractions promote speciation and the coexistence of tree species in the tropics, it ignores many important aspects of tropical rainforests, such as plant–pollinator mutualisms and phylogenetic constraints that act on trees and animal seed dispersers [29]. A more encompassing approach will necessarily consider the persistence and evolutionary branching patterns of mutualisms between angiosperm trees and their associated pollinators and seed dispersers. Tree phenology may also be important, as observed, for example, in mass fruiting events occurring in the Southeast Asian dipterocarp rainforests [73], as well as spatial limitations imposed by the complex plant architecture of the rainforest [72]. The population structure of seedlings probably reflects the movements of animal seed dispersers. These potential refinements may all affect the quantitative predictions of the nature of tree diversity, yet we expect our models to yield the same qualitative results. We find further support for our hypothesis in studies of the radiation of birds and insects in tropical regions [53,56,74,75]. Note that the extreme diversification of angiosperms in tropical rainforests evidently occurred over a fairly limited span in the Late Cenozoic, which was marked by abrupt cycles of glacial and interglacial periods over the past 2 Myr [76–78]. Our model may be expanded to include the plants with seeds spread by wind dispersal or gravity dispersal.

Our ecological simulation results imply that the coexistence of an extremely high number of tree species is possible in a relatively small geographical area (figure 2). Moreover, the results of the evolutionary simulation suggest that the final number of species depends mostly on repeated geographical isolations during the Pleistocene (figure 3). Here, the tropical forest region is separated and isolated into three or more areas, and the number of species becomes tripled or multiplied several times at each isolation event [57]. Hence the resulting number of species may be represented as *n*^*m*^, where *n* represents the number of isolated habitat areas and *m* the number of isolation events. Our hypothesis is thus consistent with the pattern of explosive speciation documented in angiosperms in tropical forests [79–81].

There are two major classes of hypotheses explaining the extremely high species diversity of trees in tropical rainforests: (i) the niche hypotheses, such as the Janzen–Connell hypothesis [4,5,19–24], and (ii) the neutral theory of communities [6,26]. Our model can incorporate the negatively density-dependent properties inspiring the Janzen–Connell hypothesis [4,5,19–24], as follows. In the current model, animal movement is purely random; therefore, the distribution of each plant species is also random. However, animals often move away from trees where they harvest fruits. Furthermore, when animals release faeces that include fruit seeds, they may avoid (or are already distant from) the parental tree, thus seedlings are likely to be dispersed away from the natal trees. We should also note that the Janzen–Connell hypothesis itself may be incorporated into the current model. Seed mortality can be increased near the cells of parental trees in the current model. By incorporating the negative density effects of seedling survival, seed predation, pathogens and the species-specific animal dispersal of seedlings, we may approach a plausible explanation for the extreme high tree species diversity of tropical rainforests. The neutral theory of communities may act as an additional factor in the temporal diversity of tree species, reducing the extinction process of new tree species with their own partner animals [6,26].

Competition and coexistence are central to the origins and maintenance of extremely high levels of tree diversity in tropical rainforests. The theory of niche separation [7] indicates that, at most, only a few tree species can coexist in a single habitat. We suspect that all animal dispersers are distinguished by possession of unique niche separations; this quality, in turn, is expected to promote the coexistence of trees. Speciation of tree species can be achieved by a slight degree of differentiation in animal movements that guarantees the coexistence of closely related tree species (figure 3*c*,*d*). Hence, as long as these animals occupy distinctive niches, various tree species may be able to coexist in the same habitats. Thus the current theory is essentially a niche theory [4,5], not so much of plants but of animal mutualists. Similarly, coexistence is also impossible in a single-layer lattice model under global interactions (electronic supplementary material, figure S1). Thus, the coexistence and persistence of many tree species can be maintained by the presence of species-specific seed dispersers. We may expand this simple model to include the mortality of animal dispersers, tree species with wind and gravity dispersal, such as Dipterocarpaceae, and the coevolution of insect pollinators to reach a more precise picture of the community biodiversity in tropical rainforests. We should stress that our model shows only one possible mechanism for extreme tree diversity and is limited by many strict conditions not necessarily applicable to tropical rainforests in general. We hope, however, that these ideas trigger various new approaches towards understanding species richness in tropical rainforests.

## Supplementary Material

Text S1

## Supplementary Material

Text S2

## Supplementary Material

Figure S1

## Data accessibility

The template of simulation programs is provided in the electronic supplementary material (Text S1 and S2).
